# A Scoping Review of Toolkits Addressing Ethical Issues in Health Registry and Clinical Research Enrollment Among African Americans With CKD

**DOI:** 10.1016/j.xkme.2026.101403

**Published:** 2026-05-12

**Authors:** Henry Asante Antwi, Adaeze Aroh, Deidra C. Crews, Rodney Elliot, Nicole Huillca, C. Daniel Mullins, Mehar Sandhu, Abdou Simon Senghor

**Affiliations:** 1 Department of Practice, Sciences and Health Outcome Research, UMB, School of Pharmacy, Baltimore, MD; 2 Beaver College of Health Sciences, Appalachian State University, Boone, NC; 3 Department of Public Health, Slippery Rock University of Pennsylvania, Slippery Rock, PA; 4 Division of Nephrology, Department of Medicine, Johns Hopkins University School of Medicine, Baltimore, MD; 5 Department of Public Health, University of Maryland, College Park, MD

**Keywords:** Chronic kidney disease, health registries, health disparities, African American, clinical research ethics, participation, toolkit, community engagement

## Abstract

Chronic kidney disease (CKD) disproportionately affects African Americans, yet they remain underrepresented in health registries and clinical research enrollment. This scoping review maps and describes existing toolkits designed to address the ethical challenges that contribute to this disparity. We conducted a scoping review following the Joanna Briggs Institute methodology. We systematically searched Ovid MEDLINE, EMBASE, Scopus, and Cochrane Central for English-language studies (2000-2024) describing tools, strategies, or frameworks to improve the ethical recruitment and retention of African American patients in CKD health registries and clinical research. Of 149 records screened, 14 US-based studies met inclusion criteria. We identified several toolkit strategies: community engagement and educational materials (eg, for APOL1 testing), educational programs (eg, for living donor transplantation), participant experience surveys, virtual recruitment tools, and enhanced genetic testing panels. Although these toolkits aimed to mitigate challenges like mistrust and complex informed consent, significant gaps in design, evaluation, and scalability were identified. Most toolkits were not rigorously evaluated for effectiveness, were often context-specific (eg, limited to transplantation), and rarely addressed structural drivers of inequity. Few provided scalable, culturally tailored solutions for the digital divide or offered sustainable models for community partnership. Although several promising toolkits exist to address ethical challenges in CKD research with African Americans, they remain nascent. Future work must focus on developing rigorously evaluated, scalable, and codeveloped toolkits that move beyond describing barriers to providing actionable, ethical solutions for inclusive registry enrollment and research participation.

Chronic kidney disease (CKD) represents a profound and persistent public health crisis in the United States-one that disproportionately and unjustly burdens African American communities. Although African Americans comprise only about 13% of the US population, they account for nearly 35% of all patients with kidney failure, reflecting a deep and ongoing failure in health equity.^1^ The disease also progresses more aggressively in this population. African Americans develop kidney failure at higher rates and transition more rapidly from early CKD stages to end-stage kidney disease, resulting in substantially higher morbidity and mortality.^2^ Although high-risk variants in the APOL1 gene increase biological susceptibility, the dominant, empirically validated drivers remain structural and clinical inequities-namely, the higher prevalence of hypertension and diabetes, economic disadvantage, environmental exposures, and persistent gaps in access to continuous, high-quality care.^3^^,^^4^ These interlocking barriers delay diagnosis, hinder disease management, and accelerate progression toward kidney failure. However, the crisis extends beyond clinical care into the realm of health registry enrollment and research inclusion**-**a critical but often overlooked driver of disparity. African Americans remain starkly underrepresented in CKD registries, biobanks, and clinical trials.^5^^,^^6^ This exclusion perpetuates inequities by weakening the very evidence base that guides clinical practice. When the populations most affected by CKD are not equitably represented in studies, the resulting findings lack generalizability, and the therapies, dosages, and management guidelines derived from them may be less effective or even unsafe for those communities.^7^

This cyclical inequity-disease burden without representation undermines both scientific validity and justice in health care. The roots of this underrepresentation lie in both historical trauma and contemporary systemic bias. The legacy of exploitation-most notoriously the US Public Health Service Syphilis Study at Tuskegee-has left deep scars of mistrust in medical research among African Americans.^8^, ^9^, ^10^ That mistrust is reinforced today by lived experiences of implicit bias, cultural insensitivity, and unequal treatment within health care and research settings.^11^ Consequently, invitations to participate in health registries or clinical studies are often met with skepticism or fear-rational responses given historical and ongoing inequities.^12^ Beyond this central issue of trust, researchers face a cluster of interconnected ethical challenges that are particularly acute for this population. The process of obtaining truly authentic and informed consent is fraught with complexity, especially in the context of genetic research. Ensuring that participants fully comprehend the potential implications, risks, and the meaning of results like APOL1 status requires more than a standard consent form; it demands culturally competent communication, ample time for questions, and a deep respect for participant autonomy.^13^

Despite extensive documentation of these barriers, practical guidance for researchers remains limited. Toolkits-curated collections of evidence-based strategies, educational materials, and implementation frameworks-offer a promising approach.^14^ They can provide step-by-step guidance for researchers and institutions to build trust with communities, facilitate enrollment in health registries and clinical research, simplify complex consent processes, reduce logistical burdens, and ensure research is conducted in a culturally respectful and equitable manner. However, no systematic assessment exists of toolkits specifically designed to support African American enrollment in CKD registries and clinical studies. It remains unknown which tools exist, what ethical challenges they target, how effectively they have been implemented, and in which critical gaps persist.^7^^,^^13^ This scoping review therefore seeks to fill this gap by systematically identifying, mapping, and evaluating toolkits or structured resources aimed at overcoming ethical challenges to African American participation in CKD health registries and clinical research. Specifically, the review will address:

1. What types of toolkits or frameworks have been developed to enhance equitable research participation?
2. What core components and ethical issues do they address?
3. What gaps remain that hinder widespread implementation?

By synthesizing and evaluating the current landscape, this review aims to guide future efforts toward building actionable, evidence-based tools that can translate the principles of ethical inclusion into practice-ensuring that African Americans with CKD are not only counted in health registries but also represented in the science that shapes their care.

## Method

We conducted this scoping review in strict accordance with the established methodology outlined by the Joanna Briggs Institute (JBI) for scoping reviews.^15^ This framework provides a rigorous, systematic, and transparent approach for mapping the breadth of literature in a field of interest, particularly in which the research is diverse in methodology or concept, as is the case with this review. The review’s reporting adheres to the Preferred Reporting Items for Systematic reviews and Meta-Analyses extension for Scoping Reviews checklist to ensure comprehensive and clear communication of our methods and findings.^16^ To ensure methodological rigor and prevent reporting bias, a detailed review protocol was developed a priori. This protocol explicitly defined research questions, objectives, and all methodological procedures, including the search strategy, eligibility criteria, and data extraction plan. In adherence to open science principles, the protocol is being formally registered on the Open Science Framework to guarantee transparency and reproducibility.

### Search Strategy and Information Sources

To capture a comprehensive and representative body of literature, we employed a systematic search strategy developed in close collaboration with a specialized health sciences librarian from the Health Sciences and Human Services Library at the University of Maryland, Baltimore. This collaboration was crucial for refining search terms and ensuring optimal retrieval across disparate databases. The search was executed across 4 major electronic databases chosen for their relevance to biomedical and public health research: Ovid MEDLINE, EMBASE, Scopus, and the Cochrane Central Register of Controlled Trials. The search timeframe spanned from January 1, 2000, to March 14, 2024, to capture the modern era of research ethics and toolkit development.

The search strategy was constructed around 3 core conceptual domains pertinent to our research question: (1) the population and condition of interest, using terms such as “chronic kidney disease,” “CKD,” “renal insufficiency,” and “kidney failure”; (2) the specific population focus, with keywords including “African Americans,” “Black,” “minority groups,” and “health disparities”; and (3) the intervention and context, using terms like “toolkit,” “framework,” “guideline,” “ethics,” “informed consent,” “community participation,” and “research enrollment.” These concepts were combined using the Boolean operators "AND" and “OR” to create a sensitive and specific search string.^15^ For databases using controlled vocabularies, such as MEDLINE (with Medical Subject Headings, or MeSH) and EMBASE (with Emtree), relevant terms were incorporated. The full, reproducible search strategy for the Ovid MEDLINE database is provided in the Supplementary Material, Item S1.

### Eligibility Criteria

Studies were selected for inclusion based on a predefined set of criteria designed to align precisely with the review’s objective. The inclusion criteria were as follows:

Publication Language: The articles published in the English language, due to resource constraints for translation.

Intervention Focus: Studies that described, developed, implemented, or evaluated a specific tool, toolkit, framework, strategy, or set of guidelines. The key requirement was the presence of a structured, actionable resource, not merely a discussion of principles or barriers.

Context: The tool must have been explicitly focused on addressing ethical issues, challenges, or dilemmas within the context of health registry or clinical research enrollment. This included, but was not limited to, interventional trials, observational studies, and cohort studies.

Population: The research must have involved, or been explicitly intended for, African American adult populations (aged 18 years or older) with diagnosed CKD or who were at elevated risk for CKD.

Geographic Scope: Studies conducted within the United States, given the specific historical, social, and health care system contexts that shape the experiences of African Americans in this country.

Conversely, studies were excluded if they: (1) only described ethical challenges or disparities without proposing or evaluating a specific interventional tool or strategy; (2) focused solely on patient education for clinical self-management without a direct link to research participation; (3) were editorials, commentaries, or narrative reviews that did not present original data or a novel toolkit; or (4) focused exclusively on health registry enrollment in which the primary activity was passive data collection without additional interventional or burdensome research procedures.

### Study Selection Process

**Figure 1:**
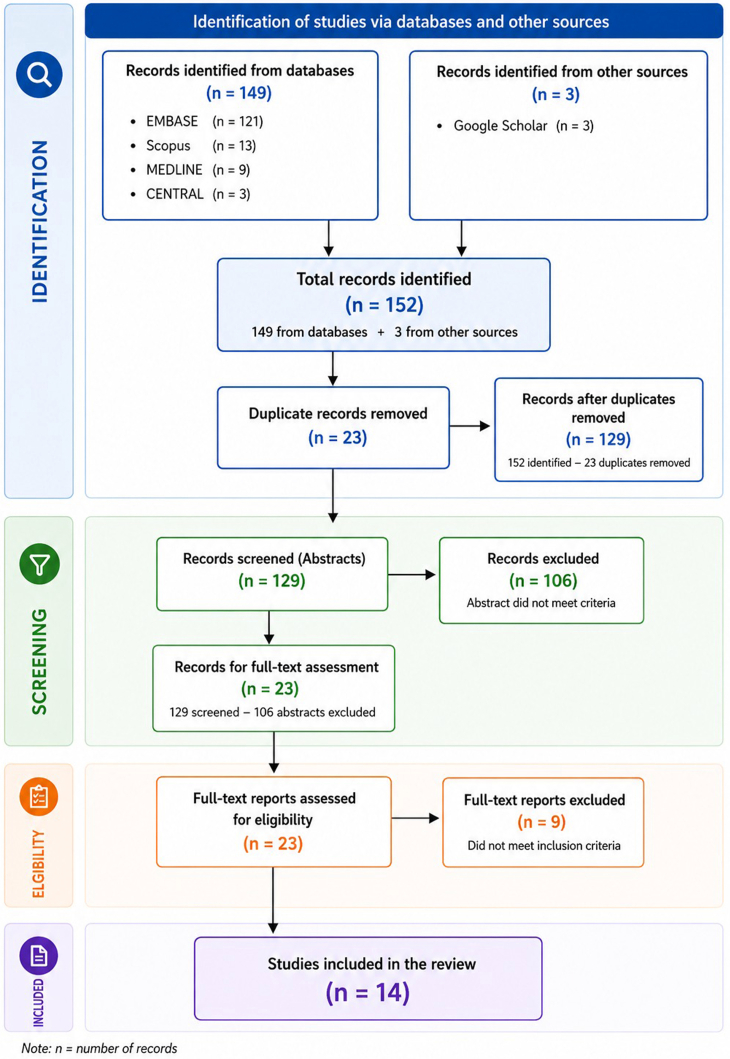
PRISMA diagram of selection process for included records in scoping review.

The management and screening of all identified records were conducted using the Covidence systematic review software, a platform designed to streamline the review process. Following the database searches, all retrieved citations were imported into Covidence, which automatically identified and removed duplicate records. The subsequent selection process was performed by 2 independent reviewers to minimize the risk of error and bias. The process began with a title and abstract screening phase, in which reviewers assessed all unique records against the eligibility criteria. Any study deemed potentially relevant by either reviewer advanced to the second stage: a full-text review. In this stage, the same 2 reviewers independently assessed the complete text of each article to make a final inclusion or exclusion decision. At both screening stages, any disagreements between the 2 reviewers were resolved through a structured discussion aimed at reaching a consensus. If consensus could not be achieved, a third, senior reviewer was consulted to adjudicate and make the final determination. The complete study selection process, including the number of records identified, screened, and included or excluded at each stage, is detailed in a PRISMA flow diagram (Fig 1).

### Data Extraction and Charting

For all studies that met the inclusion criteria following the full-text review, data were systematically extracted using a standardized, piloted data extraction form developed within Covidence. The form was piloted on a sample of 3 included studies by the review team to ensure consistency, clarity, and comprehensiveness before full-scale data extraction commenced. The data extraction was performed independently by 2 reviewers to ensure accuracy. The following key information was extracted from each included study:

• Citation details: authors, year of publication, and article title.
• Study characteristics: Primary study design (eg, randomized controlled trial, qualitative study, and review), stated objectives, and methodological description.
• Toolkit description: The name of the tool or toolkit (if provided), a detailed description of its core components and functionalities, its stated purpose, and its development process.
• Targeted ethical issue: The specific ethical challenge(s) the tool was explicitly designed to mitigate (eg, informed consent, mistrust, equitable recruitment, management of incidental findings, and participant burden).
• Stakeholders: The main stakeholders or intended audience for the tool (eg, researchers, clinicians, institutional review board members, community health workers, patients, or policymakers).
• Reported outcomes or evaluation: Any mentioned or empirically measured outcomes related to the toolkit’s use, feasibility, or acceptability.

### Quality Assessment of Individual Studies

Although formal critical appraisal to determine the risk of bias is not a mandatory component of a scoping review whose primary aim is to map the literature, we recognized the value in assessing the general characteristics and reporting quality of the included studies to contextualize our findings. Therefore, we conducted a quality assessment using a tailored approach based on the JBI critical appraisal tools. Given the methodological diversity of the included studies (eg, reviews, qualitative studies, and surveys), we selected the appropriate JBI checklist for each study design. The assessment focused on key domains relevant to the study’s design, such as the clarity of the research question, appropriateness of the methodology, detailed description of the intervention (the toolkit), and the rigor of data analysis and interpretation. This assessment was performed by one reviewer and verified by a second. The purpose was not to exclude studies based on quality but to provide a descriptive summary of the methodological landscape and to inform our narrative synthesis, allowing us to comment on the overall strength of the evidence base for the toolkits identified.

### Data Synthesis and Analysis

Given the anticipated heterogeneity in the designs of the included studies and the nature of the toolkits themselves, a meta-analysis was neither appropriate nor feasible. Instead, we employed a narrative synthesis approach to summarize and explain the findings. The synthesis process involved several stages. First, we used descriptive statistics (frequencies and proportions) to summarize the general characteristics of the included studies, such as publication years, study designs, and geographical locations. Second, we conducted a thematic analysis to inductively categorize the identified toolkits based on their primary strategy or mechanism of action (eg, educational, technological, or community engagement). Finally, we created a narrative summary and structured tables to map these toolkit categories against the key ethical challenges they aimed to address, their intended stakeholders, and the reported gaps or limitations, thereby providing a comprehensive overview of the current state of the field.

## Results

The PRISMA flow diagram (Fig 1) outlines the study selection process. The initial search yielded 149 records. After screening, 14 studies met the inclusion criteria.

### Characteristics of Included Studies and Toolkits

The 14 studies included in this review were published from 2009 to 2024 and employed a range of study designs. These included primary observational studies, cross-sectional survey studies, quasi-experimental interventions, case series, and qualitative stakeholder-engagement studies (n = 10), as well as narrative and conceptual reviews (n = 4). Specifically, the review incorporated multisite cohort studies,^17^ empirical ethics studies,^18^ cross-sectional observational survey studies,^3^^,^^19^^,^^20^ quasi-experimental intervention studies,^9^ case series with conceptual analysis^21^ and qualitative stakeholder-engagement studies.^22^ Conceptual and narrative reviews included discussions on Indigenous populations and ethical frameworks in kidney disease research.^23^, ^24^, ^25^, ^26^ All studies were conducted in the United States or involved US populations. The identified toolkits and strategies were subsequently categorized into 5 primary types (Table 1).^3^^,^^8^^,^^9^^,^^13^^,^^17^, ^18^, ^19^, ^20^, ^21^, ^22^, ^23^, ^24^, ^25^, ^26^, ^27^

**Table 1:** Toolkit Categories and Characteristics

Toolkit Category	Description & Examples	Targeted Ethical Challenge(s)	Key Gaps Identified
Community engagement & education (Tuttle et al,^17^ [2021]; Alnaes,^18^ [2012]; Young et al,^22^ [2019])	Stakeholder meetings, deliberative forums, and culturally tailored educational materials (eg, for APOL1 testing).	Mistrust, inadequate informed consent, and lack of cultural relevance.	Limited scalability; lack of long-term sustainability models; and lack of reported evaluation data on enrollment impact.
Educational programs (Waterman et al,^26^ [2010]; Gordon et al,^9^ [2016])	Home-based education, clinician training programs to address disparities in living donor kidney transplantation.	Inequitable access, lack of awareness, and implicit bias.	Resource-intensive and limited adaptation for broader CKD research recruitment.
Participant feedback mechanisms (Victoria-Castro et al,^3^ [2024]; Moledina et al,^19^ [2018]; Eneanya et al,^20^ [2016])	Surveys to assess participant experience with procedures like research kidney biopsies.	Participant burden, anxiety, improving respect and beneficence.	Reactive rather than proactive and does not address initial trust barriers.
Virtual recruitment tools (Sohansoha et al,^27^ [2023])	Online platforms and digital strategies for recruiting patients into clinical trials.	Geographic and logistical barriers to access.	Risks exacerbating the digital divide and may not build necessary interpersonal trust.
Enhanced genetic protocols (Mohanasundaram and Fernando,^24^ [2023]; Young et al,^22^ [2019]; Singh et al,^21^ [2022]; Reese et al,^25^ [2021]; Young et al,^13^ [2017]; Cronin^8^, [2014]; Kolewaski and Yeates,^23^ [2009])	Specialized genetic testing panels and tailored informed consent processes for living kidney donors.	Complex informed consent, management of incidental findings.	Narrowly focused on transplantation contexts; not evaluated for broader CKD registry enrollment.

### Synthesis of Gaps in Current Toolkits

Our analysis revealed consistent gaps across the toolkit categories:

• Lack of evidence for effectiveness: Few toolkits were associated with empirical data on outcomes such as recruitment, retention, or equity.
• Limited scalability and sustainability: Many initiatives, particularly intensive community engagement and home-based programs, were resource-heavy with no clear path for widespread implementation.
• Insufficient address of structural barriers: Toolkits primarily focused on educating individuals or improving communication, with limited strategies to directly address systemic racism and institutional policies that hinder participation.
• Narrow contextual application: Several promising tools (eg, enhanced genetic protocols) were developed for specific contexts like transplantation and have not been adapted for the broader spectrum of CKD research.

## Discussion

This scoping review provides a systematic evaluation of toolkits designed to navigate the ethical complexities of involving African Americans in CKD clinical research. We identified a range of strategies, from community partnerships to digital recruitment, yet found that few toolkits reported empirical evidence demonstrating their impact on equitable enrollment. The current landscape of toolkits is characterized by innovation in approach but a critical shortfall in validation and scalability. For instance, while community engagement is rightly emphasized as a cornerstone for building trust the reviewed toolkits often described one-off forums rather than durable, shared-governance models that transfer real power to communities.^22^

Similarly, the push toward virtual recruitment recognizes the need for modernized methods but fails to adequately contend with the digital literacy and access barriers that may systematically exclude the most vulnerable patients, thereby creating new forms of inequity.^27^ A key gap is the lack of reported toolkit evaluation. Although interventions have been designed, few studies applied rigorous metrics to assess their implementation or outcomes. This leaves researchers and institutions without evidence-based guidance on which strategies are worth their often-substantial investment of time and resources.

### Implications for Policy, Practice, and Research

To advance the field, future efforts must be directed toward the development of next-generation toolkits that are:

1 Codeveloped and community-owned: Moving beyond consultation to models in which communities have decisional authority in research design and benefit-sharing.
2 Rigorously evaluated: Incorporating prospective, mixed-methods studies to measure toolkit impact on recruitment rates, participant trust, and ethical satisfaction.
3 Scalable and hybrid: Combining the high-touch, high-trust elements of community engagement with the broad reach of digital tools, while providing technical support to bridge the digital divide.
4 Structurally informed: Including components that address researcher’s implicit bias and advocate for policy changes within research institutions to remove systemic barriers.

We propose a conceptual framework for future toolkit development that matches specific ethical challenges with actionable, evaluable strategies, as summarized in Table 2.

**Table 2:** Proposed Framework for Future Toolkit Development

Ethical Challenge	Actionable Toolkit Strategy	Metric for Success
Historical mistrust	Establish community advisory boards with veto power on study materials and data use.	Increased community satisfaction scores; higher rates of study approval by the board.
Complex informed consent	Develop interactive, culturally tailored digital decision aids with teach-back components.	Improved scores on knowledge tests post-consent; reduced participant anxiety.
Logistical & psychological burden	Implement a patient navigation program staffed by trained community health workers.	Improved participant retention rates; reduced missed appointments.
Systemic access barriers	Create institutional policies for compensating participants for time and travel; offer childcare.	Increased enrollment from low-income populations.

### Limitations

This review has limitations. The focus on US studies limits generalizability to other contexts. As a scoping review, it synthesizes the landscape but does not critically appraise the quality of individual studies. Furthermore, the available literature may reflect a publication bias toward describing interventions rather than reporting their null or negative results.

## Conclusion

This scoping review consolidates the current state of toolkits designed to promote the ethical engagement of African Americans in CKD clinical research. It demonstrates a clear consensus on the types of strategies needed but reveals a critical gap in evaluation evidence regarding their scalability and ability to enact structural change. The findings illuminate a path forward not only for clinical trials but also for the crucial, yet often overlooked, domain of health registry enrollment. The ethical challenges of mistrust, complex consent, and logistical burdens are not unique to interventional studies; they form the same fundamental barriers to health registry enrollment. Although registry enrollment may be less burdensome, it still requires individuals to share sensitive personal data with institutions they may distrust.

Therefore, the toolkit categories identified, particularly community engagement and education and participant feedback mechanisms, are directly applicable. For instance, toolkit strategies that use community forums to explain how registry data is used, who has access, and how it benefits the community can help build the transparency necessary for trust. Similarly, simplified, tiered consent processes developed for clinical research can be adapted to make registry enrollment more understandable and accessible. The policy implications of this review extend directly to registry governance. For policymakers and registry stewards, our findings argue for a proactive approach. This includes:

• Funding and mandating community co-governance of registries, ensuring African American communities have a decisive voice in data use, access policies, and the communication of aggregate results.
• Developing and validating registry-specific toolkits that address the unique consent and engagement challenges of longitudinal data collection, moving beyond the one-time, lengthy consent form to dynamic, ongoing communication.
• Creating policy that requires and funds the evaluation of enrollment strategies for public health registries, ensuring they do not passively perpetuate the same disparities present in clinical trials.

The underrepresentation of African Americans in health registries creates a flawed data foundation, skewing our understanding of CKD’s natural history, treatment effectiveness, and outcomes across the entire population. By applying the lessons from this review, specifically, the need for co-created, evidence-based, and structurally-aware strategies, we can begin to build more equitable and representative data infrastructures.

## Supplementary Materials

Supplementary File (PDF) Item S1
