# Supplementary material for: A Scoping Review of Toolkits Addressing Ethical Issues in Health Registry and Clinical Research Enrollment Among African Americans With CKD
Source: Kidney Med. 2026 May 12;8(7):101403. doi: 10.1016/j.xkme.2026.101403 (PMC13312113; doi:10.1016/j.xkme.2026.101403)
Supplement: Supplementary File (PDF) — Item S1 [file mmc1.pdf]

Item SI. Ovid MEDLINE Search Strategy

Database: Ovid MEDLINE (ALL)
Date Range: January 1, 2000 – March 14, 2024
Language: English
Population: Adults (≥18 years)
Geography: United States

A. Combined Ovid MEDLINE Search Strategy (Journal-Ready)

(
 exp Chronic Kidney Disease/
 OR exp Renal Insufficiency, Chronic/
 OR exp Kidney Failure, Chronic/
 OR (chronic kidney disease OR CKD OR chronic renal disease OR renal insufficiency OR kidney failure).ti,ab.
)
AND
(
 exp African Americans/
 OR exp Minority Groups/
 OR exp Health Disparities/
 OR (African American* OR Black American* OR Black population* OR racial minorit* OR ethnic minorit*).ti,ab.
 OR (health disparit* OR racial disparit* OR inequ* OR underserved population*).ti,ab.
)
AND
(
 exp Ethics, Research/
 OR exp Informed Consent/
 OR exp Patient Participation/
 OR exp Community Participation/
 OR exp Research Subjects/
 OR (research ethic* OR ethical issue* OR ethical challenge*).ti,ab.
 OR (informed consent OR research participation OR research enrollment OR clinical trial enrollment).ti,ab.
 OR (community engagement OR stakeholder engagement).ti,ab.
)
AND
(
 exp Practice Guidelines/
 OR exp Guideline Adherence/

OR exp Program Development/
OR exp Models, Theoretical/
OR (toolkit* OR tool kit* OR framework* OR guideline* OR strategy OR strategies OR intervention* OR model*).ti,ab.
OR (best practice* OR implementation strateg* OR decision aid*).ti,ab.
)

Limits: English language; Humans; Adults (≥18 years); United States.
Search period: January 1, 2000 – March 14, 2024.

Note: Both controlled vocabulary (MeSH) and free-text terms were used; the strategy was developed in collaboration with a health sciences librarian.

B. Line-by-Line Ovid MEDLINE Search Strategy (PRISMA-S Compliant)

1. exp Renal Insufficiency, Chronic/

2. exp Kidney Failure, Chronic/

3. exp Chronic Kidney Disease/

4. (chronic kidney disease or CKD or chronic renal disease or renal insufficiency or kidney failure).ti,ab.

5. 1 or 2 or 3 or 4

6. exp African Americans/

7. exp Minority Groups/

8. exp Health Disparities/

9. (African American* or Black American* or Black population* or racial minorit* or ethnic minorit*).ti,ab.

10. (health disparit* or racial disparit* or inequ* or underserved population*).ti,ab.

11. 6 or 7 or 8 or 9 or 10

12. exp Ethics, Research/

13. exp Informed Consent/

14. exp Patient Participation/

15. exp Community Participation/

16. exp Research Subjects/

17. (research ethic* or ethical issue* or ethical challenge*).ti,ab.

18. (informed consent or research participation or research enrollment or clinical trial enrollment).ti,ab.

19. (community engagement or community participation or stakeholder engagement).ti,ab.

20. 12 or 13 or 14 or 15 or 16 or 17 or 18 or 19

21. exp Practice Guidelines/

22. exp Guideline Adherence/

23. exp Program Development/

24. exp Models, Theoretical/

25. (toolkit* or tool kit* or framework* or guideline* or strategy or strategies or intervention* or model*).ti,ab.

26. (best practice* or implementation strateg* or decision aid*).ti,ab.

27. 21 or 22 or 23 or 24 or 25 or 26

28. 5 and 11 and 20 and 27

29. limit 28 to English language

30. limit 29 to humans

31. limit 30 to adults (18 years and older)

32. limit 31 to United States

33. 32
